# What Is Being Used and Who Is Using It: Barriers to the Adoption of Smartphone Patient Experience Surveys

**DOI:** 10.2196/formative.9922

**Published:** 2019-03-18

**Authors:** Denise Ng, Josephine McMurray, James Wallace, Plinio Morita

**Affiliations:** 1 School of Public Health & Health Systems Faculty of Applied Health Sciences University of Waterloo Waterloo, ON Canada; 2 Business Technology Management Lazaridis School of Business & Economics Wilfrid Laurier University Brantford, ON Canada

**Keywords:** quality of healthcare, surveys and questionnaires, patient satisfaction, data collection, smartphone, mobile phone, risk, privacy

## Abstract

**Background:**

Smartphones are positioned to transform the way health care services gather patient experience data through advanced mobile survey apps which we refer to as smart surveys. In comparison with traditional methods of survey data capture, smartphone sensing survey apps have the capacity to elicit multidimensional, in situ user experience data in real time with unprecedented detail, responsiveness, and accuracy.

**Objective:**

This study aimed to explore the context and circumstances under which patients are willing to use their smartphones to share data on their service experiences.

**Methods:**

We conducted in-person, semistructured interviews (N=24) with smartphone owners to capture their experiences, perceptions, and attitudes toward smart surveys.

**Results:**

Analysis examining perceived risk revealed a few barriers to use; however, major potential barriers to adoption were the identity of recipients, reliability of the communication channel, and potential for loss of agency. The results demonstrate that the classical dimensions of perceived risk raised minimal concerns for the use of smartphones to collect patient service experience feedback. However, trust in the doctor-patient relationship, the reliability of the communication channel, the altruistic motivation to contribute to health service quality for others, and the risk of losing information agency were identified as determinants in the patients’ adoption of smart surveys.

**Conclusions:**

On the basis of these findings, we provide recommendations for the design of smart surveys in practice and suggest a need for privacy design tools for voluntary, health-related technologies.

## Introduction

### Background

High-quality patient-centered care is widely recognized as a priority in health care [1] and has been shown to improve patient experience, patient safety, and accessibility to services [2,3]. Health care professionals engaged in patient-centered care focus on ensuring that patients’ experiences at a physician, hospital, or rehabilitation facility meet basic standards of care, such as treating them with courtesy, informing them about their care, and minimizing pain during their visit. Standardized questions surrounding these aspects of a medical visit are widely used within the health care industry to inform patient-centered care best practices and to improve governance, public accountability, and patient autonomy [4,5].

Paper-based collection of experience surveys remains time-consuming, expensive, and limited by factors such as nonresponse, recall bias, and inadequate sample size [6-8]. Although paper-based surveys may be advantageous in some circumstances, that is, to reduce startup costs or for nondigital natives such as the elderly, the use of smartphone-based survey apps, which we call *smart surveys*, provides new opportunities to improve data collection techniques. Mobile technology overcomes many of the limitations of paper-based surveys and enables collection of large quantities of real-time data over a broad geographical area. Exploiting this technology opens the possibility of private and public sector services, health care providers, and government bodies effectively engaging with the public, one-on-one, to better respond to their needs. However, at present, there is little guidance to help service providers understand when and where individuals are willing to disclose service experience data using their smartphones. In particular, there is a lack of understanding of users’ beliefs, perceptions, and attitudes toward sharing of health service experience feedback using their smartphones.

### Patient Experience Surveys

Patient experience surveys are validated questionnaires, which are developed by health services experts to understand patient perceptions of their health care experience and serve an integral role in patient engagement and service improvement [3]. For example, the WatLX patient experience questionnaire [9-11] determines agreement with statements such as “I was always treated with courtesy,” “My physical pain was controlled as well as possible,” or “From now on I know what to expect about my care.” When such patient experience feedback is collected, collated, and interpreted, findings can drive critical and necessary improvements in service quality, patient safety, and clinical effectiveness [3,4]. In recent years, to increase the benefits yielded from such surveys, health service research has begun to shift from “traditional” paper-based methods of survey administration to the use of technology-enabled survey tools. Yet there is little published research on the use of smartphone technology to collect individuals’ experiences of health services.

The health care literature has found no significant differences in data equivalence or validity between paper- and Web-based surveys [12-14]. With mobile devices, researchers are able to collect large quantities of data over broad geographical areas. Furthermore, the use of smartphone-based apps provides access to functionality such as location-based activity detection [15,16] and notifications [17] that can help improve survey compliance and completeness of survey responses to improve the overall reliability of results [18]. In situ assessment of services, where data are collected during or immediately after a service encounter, may yield an even more immediate and insightful level of understanding of service quality [19]. However, the context in which these data are collected can also introduce new issues such as concerns around privacy [20].

Smart surveys, which we define as smartphone survey apps that use advanced functionality to provide more intuitive surveys or gather more contextual complementary data in addition to participant responses, introduce a number of unfamiliar behaviors for patients to undertake that may work as barriers to adoption. For example, they require users to download an app to their personal smartphone and to disclose potentially sensitive information via a digital channel that may be perceived as public or insecure. To better identify and understand how these barriers may impact the adoption of smart surveys, we turn to the theory of perceived risk [21,22].

### Perceived Risk Theory

A user’s perceptions of risk can have a negative effect on information system (IS) adoption [21,23,24]. Initially introduced in the context of consumer behavior research, perceived risk can be conceptualized as the subjective expectation of loss experienced by a consumer during purchase decisions [21]. Perceived risk describes risk as a multidimensional construct that comprises facets such as financial risk, performance risk, physical risk, psychological risk, social risk, time risk, and privacy risk. Our framework of perceived risk is adapted from studies by Jacoby and Kaplan [22] and Featherman and Pavlou [23] (Table 1).

**Table 1:** Seven dimensions of perceived risk framework.

Risk dimension	Definition
Performance	The possibility that a product or service is not performing the way it was designed or advertised, therefore failing to deliver the expected benefits.
Financial	The possibility that the use of a product or service will cause undesired financial loss (due to purchase and incurring fees or fraud).
Time	The possibility that a product or service will cause the consumer to lose time from researching the product, learning the use, or returning the product if it underperforms.
Psychological	The risk that the purchase or performance of a product or service will cause a negative effect on the consumer’s mind or self-perception (eg, frustration or loss of self-esteem).
Social	The potential loss of the consumer’s social circle due to the use of a product or service.
Physical	The possibility that the use of a product or service may be harmful or injurious to the consumer’s health.
Privacy	The potential for personal information being shared without consent and/or used for purposes other than originally intended.
Overall	A general measure of perceived risk when all criteria are considered together.

Consumer behavior and IS research has found perceived risk and its antecedents to be key predictors of electronic service adoption; for example, perceived risk and its dimensions are inhibitors of technology acceptance model variables [19]. Of all the facets of perceived risk, privacy (security) risk is demonstrated to be the most important barrier in the adoption of e-services for consumers, having both direct and indirect influences on the intention to adopt [25]. Financial risk, the second most important inhibitor to adoption, also has a significant negative impact on attitude to technology adoption [26]. Time risk has a negative influence on attitudes, implying consumers are concerned about delays and length of time to complete a transaction.

Perceived risk impacts attitudes toward adopting mobile e-services [27-29] as well as the intentions of use among both frequent and infrequent users of mobile e-services. In these studies, results have consistently shown overall perceived risk to be mediated by privacy, financial, time, and performance risks. With respect to mobile health (mHealth) app adoption studies, perceived risk has significant and negative effects on attitudes toward adoption. For example, Schnall et al [30] identified patient concerns regarding security (eg, health information or location sharing) when referring to mHealth apps and smartphone devices, similar to Zhou [31].

Previous research has categorized users based on the intensity of their perceptions of privacy risk. Westin [32] separates technology users into 1 of the 3 risk groups based on their willingness to share personal information on the Web: (1) privacy fundamentalists (high privacy orientation and supports regulatory controls), (2) privacy pragmatists (weigh benefits to self or society and bases trust on context), and (3) privacy unconcerned (willing to share information and reject privacy concerns). These categories were developed over a series of more than 30 consumer surveys [33] and have been used extensively by the computer-human interaction community as a requirements engineering design tool to help anticipate user needs and design functionally relevant technology. However, in practice, this methodology has faced criticism, and research has found a lack of correlation between Westin’s categories and user behaviors and attitudes (ie, willingness to share information), perhaps attributable to the development of the questions before the internet [34-36]. In response to this mismatch between Westin’s categories and user behavior and to more fully account for the importance of privacy risk as a barrier to adoption of smart surveys, we turn to Dupree et al’s work on privacy personas [37,38].

### Privacy Personas

Dupree et al [37] developed privacy personas to add contextual information to participants clustered by their attitudes and behaviors related to security and privacy. The personas provide a better understanding of a user’s proactivity and ability to act upon privacy risk concerns, better aligning their behavior with their attitudes. Dupree et al identify the following 5 clusters:

1. Fundamentalists (high knowledge and motivation): like Westin’s privacy fundamentalists [33], these individuals are highly concerned with privacy and show distrust toward corporate monitoring. They exercise extreme caution when handling their information, often encrypting their devices.
2. Lazy experts (high knowledge and low motivation): these individuals share the same technical knowledge as fundamentalists, but often choose convenience over security and socialization over privacy. They continue to put effort into protecting their privacy, however not to the extent where they would limit their interactions with society.
3. Technicians (medium knowledge and high motivation): have less technical knowledge compared with the fundamentalists and lazy experts. However, they show limited trust in privacy settings and are highly motivated to protect their privacy, often choosing privacy over being social. They tend to form their attitudes more intuitively but will change their behavior when provided with evidence.
4. Amateurs (medium knowledge and medium motivation): these individuals are just learning about security concepts. They are not nearly as motivated or knowledgeable as the other previously mentioned groups. Despite having limited knowledge, this group will still act to protect themselves from privacy threats.
5. Marginally concerned (low knowledge and low motivation): with limited knowledge about security concepts, they trust networks and websites which claim to be safe. They are aware of potential privacy threats but feel these threats are unlikely to happen to them.

Morton and Sasse [39], who performed their research concurrently with Dupree et al, also identify 5 clusters that closely correspond to those listed above but in the context of location disclosure.

The purpose of this study was to understand what beliefs, perceptions, and attitudes influenced patients’ intentions to share health service experience feedback using their smartphones, in particular, what role perceived risk plays in this process. Health care providers are increasingly being held accountable for the quality of services they provide; however, data collection is expensive, response rates are low, and turnaround times can be long. Although mHealth apps are common in the sector, and smartphones have been used to collect experience data in other industries [40,41], there has been little research into the use of mobile apps to collect in situ, location-based experience data in health care.

## Methods

### Participant Recruitment and Selection

We recruited participants from a local university between January and February 2017 using posters, email, and snowball sampling techniques. Participants were classified according to their privacy persona and their dimensions of perceived risk, and their responses were sequentially analyzed to allow researchers to evaluate the breadth of our sample and to ensure that individuals with different technical backgrounds as well as varying degrees of privacy tolerance related to information sharing were included in the study. Recruitment and analysis proceeded until saturation [42,43]. Participants received Can $10 for participating in the interview.

**Figure 1:**
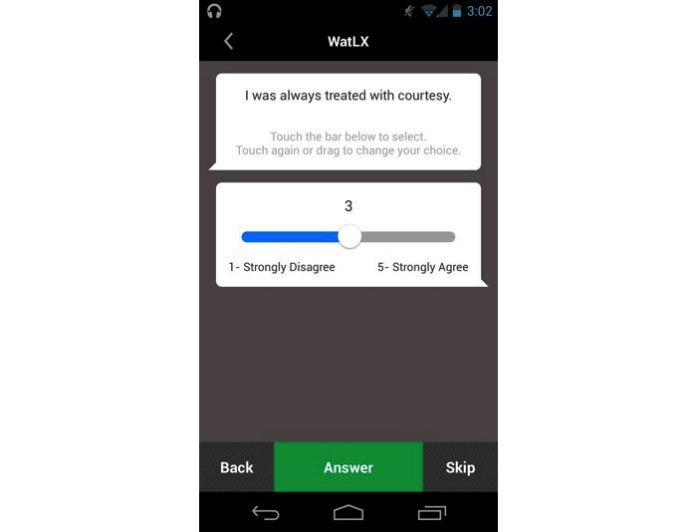
MetricWire's smartphone app served as a platform to administer the patient experience SmartSurvey.

### Data Collection and Analysis

Participants were welcomed upon their arrival and were given an overview of the purpose of the study and the data collection process by a researcher. Participants reviewed and signed an informed consent form and provided demographic information. Data were collected from participants using short questionnaires followed by a semistructured, in-depth interview. The information gathered from the questionnaires and think-aloud technique provided information for classification of participants into privacy persona clusters as well as complementary data for further context.

In the first questionnaire, participants were asked to rate their perspectives on privacy and security (PAS) (Multimedia Appendix 1). The second questionnaire included Westin’s privacy user questions [32] and questions that were related to their knowledge and motivation to protect their privacy (Multimedia Appendix 2) [37]. Before completing the final questionnaire and interview, participants were given the opportunity to learn more about the mobile app used for the survey distribution, analysis, and administration [44] and to evaluate its key features and user interface. The MetricWire app is a commercially available mobile phone and Web-based survey platform [44]. The app allows mobile completion of surveys, with functionality such as automatic alerts or triggers to prompt phone owners to respond to a survey based on factors such as time of day or location of the device. Researchers loaded a modified standardized and validated patient experience survey [9,10] into the MetricWire platform using a smartphone provided by the researchers (Figure 1). Participants were asked to recall their last visit to a physician and fill out the survey. Completion of the survey ranged from 2 min to 5 min.

Using cognitive interviewing techniques [45] (think aloud), where questions are administered, and participants are encouraged to verbalize the reasoning behind their answers, a third questionnaire (including questions adapted from Jacoby and Kaplan’s [22] perceived risk study), was used to assess participants’ perceptions of risk (Multimedia Appendix 3). These semistructured participant interviews ranged from 20 min to 40 min. Throughout and at the end of the interview, the researcher summarized their interpretation of each participant’s responses. Participants were encouraged to add any additional information that they felt was missing from the summarized interview responses. This process served as an informal method for member checking [46].

Upon completion of each participant interview, participant responses were transcribed manually from the digital recordings and thematically analyzed using QSR International’s NVivo 11 [47]. Responses were used to inform subsequent interviews. We used constant comparison and content analysis to code and analyze the transcripts [48], with 3 researchers (DN, JM, and JW) reviewing the interview transcripts independently and using consensus methods to iteratively discuss content and discrepancies to ensure coding consistency. Data were manually sorted using the perceived risk framework and then thematically analyzed to uncover the unique challenges facing smart surveys’ adoption and use for patient experience sampling. Interview transcripts underwent initial open-ended coding where quotes were divided into 4 concepts and 12 subconcepts based on similarities in meaning or context. These concepts were then discussed among the researchers to further develop emergent themes. The themes were developed based on both the perceived risk typology (deductive reasoning) and open and axial coding of interview transcripts (inductive reasoning) [47,49].

To assign participants to 1 of Dupree’s clusters, each participant’s data were reviewed (DN), and the participant was preliminarily assigned. Following a process of discussion (JM, JW, and PM), participants were reassigned as necessary until each cluster remained stable. Participant data were collected until we had at least one participant from each of the Dupree clusters identified, and saturation was achieved, where no new themes or evidence emerged from the interview transcripts [42,43].

Ethics approval for this study was sought and obtained jointly from the ethics committees at Wilfrid Laurier University and the University of Waterloo (#4690). All participants provided written informed consent before participating in the study.

## Results

### Overview

We conducted 24 semistructured interviews with Canadian smartphone owners (7 male and 17 female) with varying educational backgrounds, technical knowledge, and motivations to protect privacy. All the participants were registered university students, half at the graduate level and, as such, are “digital natives” and thus confident using smartphones and mobile apps [50]. All had received some postsecondary or postgraduate education, were comfortable speaking English, and regularly used a smartphone. The average age of the sample was 23.9 years (minimum: 21 years, maximum: 56 years, and median: 22.5 years).

### Perceived Risks and Privacy Personas of the Sample

Respondents were classified according to Dupree et al’s [37] privacy clusters framework (Table 2). Participant 4 was rated with *low technical knowledge* yet *high motivation* to protect their privacy. Therefore, they did not fit any of the Dupree privacy personas, and we classified them as “Undefined.”

To develop an understanding of the core issues facing smart survey adoption, we also categorized responses according to the dimensions of perceived risk [21,22] (Table 3). Participants were most concerned about PAS and performance risk; under the circumstances, that is, the introduction of a novel app for use in a contextually sensitive location, this result was predictable. None of the participants expressed concerns related to their psychological or social well-being as a result of using the app.

**Table 2:** Participants classified by privacy persona.

Privacy persona	Knowledge	Motivation	Statistics, n (%)
Marginally concerned	Low	Low	8 (33)
Technicians	Medium	Medium	7 (29)
Amateurs	Medium	Low	5 (21)
Lazy experts	High	Low	2 (8)
Fundamentalists	High	High	1 (4)
Undefined	Low	High	1 (4)

**Table 3:** Number of participants who classified dimensions of perceived risk as either “likely” or “very likely.”

Type of perceived risk	Statistics, n (%)
Privacy and security	18 (75)
Performance	12 (50)
Time	4 (17)
Financial	2 (8)
Physical	1 (4)
Psychological	0 (0)
Social	0 (0)

### Thematic Analysis

A number of themes emerged from our analysis of cognitive and in-depth interview transcripts: (1) perceived risks associated with smart survey use, (2) loss of information agency, and (3) trusted data collectors and altruistic intentions. These are organized according to the focus of this study: first, how perceived risk impacts the propensity to use smartphones to provide service feedback using our perceived risk framework, and second, the role of participants’ key beliefs, perceptions, and attitudes in that process.

#### The Impact of Perceived Risk on Intentions to Use Smart Surveys

##### Performance Risk

Although performance risk was the most cited type of risk, participants perceived it to be minimal when downloading or using smart surveys. The likelihood of performance risk was rated by 25% (6 out of 24) participants as “Very Unlikely” and as “Unlikely” by 46% (11 out of 24) participants. Some participants attributed this lack of risk to smart surveys being more simplistic in design than other apps on their phone and others to functionality that allowed participant audit before data were submitted:

...from my point of view, it doesn’t look too fancy or a gaming application with a lot of coding and stuff...I feel like chances of it not working...will be low.

P21, Amateur

It’s not quite as advanced as some other apps. And since it submits [data] all at once... I would be able to look at all the information before it’s submitted.

P14, Lazy expert

##### Time Risk

Some participants commented that smart surveys’ voluntary nature mitigated any associated risks related to time. Others disagreed, saying time loss from downloading and using the app was “very likely”; as they perceived that only health care providers would ultimately benefit from the data, they saw no off-setting personal benefit to mitigate the time risk:

Very likely, because it does benefit just the company, not really yourself. And like I said, it already takes a long time as an app it downloads and all that stuff...

P4, Undefined

##### Financial Risk

The majority of participants felt that the possibility of financial loss associated with the app was either “Very Unlikely” 71% (17 out of 24 participants) or “Unlikely” 21% (5 out of 24 participants). The perception of low financial risk was attributed to smart surveys’ free download and lack of request for any financial information:

As a patient, would I have to pay money to download the app?...In this case, there doesn’t seem like there’s any chance that I would be losing money with Smart surveys. I don’t think it’s asking for credit card information or anything.

P22, Lazy expert

##### Psychological Risk

When asked to judge their perception of psychological risk associated with smart surveys, nearly all participants 8% (20 out of 24) rated their perceived psychological risk to be “Very Unlikely.” Participants were familiar with providing feedback and with using smartphone apps:

...it’s voluntary if there was something I didn’t want to say or discuss, I wouldn’t have taken it.

P21, Amateur

##### Social Risk

Overall, participants perceived a very low possibility of social risk, noting that completing surveys on a smartphone was sociably acceptable and could be completed privately:

I’m on my phone a lot anyways. I’m answering surveys. I don’t think anyone would think of me differently because it’s just surveys.

P7, Technician

##### Physical Risk

There was little to suggest that participants perceived any physical risk associated with this technology and noted that it was comparable with any other app on their smartphone:

Well, it’s just filling out buttons on a survey. I don’t think there should be health issues any more than health issues from just using a smartphone.

P7, Technician

##### Privacy and Security Risk

In line with the 6 dimensions of risk, participants rated the likelihood of “overall” risk associated with smart surveys as low. However, when discussing the overall risk, the predominant concerns related to PAS risk included the loss or misuse of sensitive information associated with their location and activity:

Personally, I don’t like the idea of data being collected on me...If there’s an app that could literally tell you physically where you’re being, that’s part of the metadata government can collect on you.

P12, Technician

Similarly, participants displayed heightened sensitivity and apprehension about the possibility of the app being used to retrieve additional information unrelated to the research:

Maybe if I download some app, maybe someone can get your personal information on your phone.

P6, Marginally concerned

Moreover, 1 participant was concerned that a third party, such as an employer or insurance company, could use the collected data to deny individuals employment or insurance claims:

If it’s not associated with my insurance company in any way, and it’s only for the health care to improve their staff’s interaction with their patient. I don’t think it would be likely [I would consider it a risk].

P24, Technician

Yes. I just think I would just want to know what is being used and who’s using it. And if someone could tell me that, then it might change my mind from not giving out that information to giving information.

P11, Marginally concerned

I don’t consider the information to be very sensitive. Even if it does go into the wrong hands, which would be weird, I probably wouldn’t mind too much.

P7, Technician

Another expressed a belief that mobile apps may be less secure than traditional desktops apps and that the use of smartphones introduces risks such as susceptibility to hackers, in-device vulnerability, and susceptibility to loss:

It’s not 100% safe...I’m not sure apps interact with each other in a smartphone...if other apps can steal information from another app. It’s not 100% safe.

P21, Amateur

I think it’s safe. It’s not risky to share feedback. But you never know. Sometimes people can get your secure passwords, your bank passwords.

P23, Amateur

...it’s not really safe to send it through the smartphone...a smartphone can easily go into the wrong hands. It could get stolen, or even borrowed, maybe you just left it somewhere...

P7, Technician

Location (Global Positioning System, GPS) data were an area of particular sensitivity. The majority of participants 71% (17 out of 24) were reluctant to disclose their location (GPS) data for service quality improvement. Many chose to not share location information for reasons of privacy, safety, and battery life:

If it’s on all the time, I feel like someone’s following me all the time or someone can see that they’re following me and it probably drains out my battery too.

P13, Amateur

Other issues included concern over the perceived lack of standards surrounding the handling and storage of patient experience data. The heightened sensitivity was not surprising given the considerable attention to PAS risks associated with mHealth apps [51] and frequent breaches of health ISs in the free press at the time. Finally, the overall usability of smartphones relative to desktops was also considered, reflecting limitations of a smartphone’s display as part of their confidence in the device to live up to the task of completing a survey:

...I mean, it’s kind of normal for me to do surveys on a computer but doing it on the phone is a little awkward. Another reason, I guess, I’m not too fond of reading too much on a smartphone ’cause I have a smaller screen and the text is small.

P7, Technician

#### The Role of Participants’ Beliefs, Perceptions, and Attitudes

##### Trusted Data Collectors and Altruistic Intentions

Importantly, the third-party mobile app for data collection using a smartphone was perceived as distinct from the health care facility requesting the data, which participants generally trusted to comply with ethical treatment of their data:

...because it’s health care facility. I have complete trust in them.

P2, Amateur

The identity of those who receive and interpret patient experience data was an important consideration for participants when deciding to complete a patient experience smart survey. More than half the participants 6% (14 out of 24) mentioned concerns over who received and viewed their information. Knowing who the users were and how the data would be used helped them decide whether or not to share feedback. Sharing experience data with their care providers was not a barrier, given its less sensitive nature, and thus diminished consequences if mishandled.

For some participants with altruistic intentions, the *impact* of their feedback was a significant factor in decision making, particularly where trusted care providers might directly or indirectly use that feedback to improve service quality for others:

I want my feedback to improve the service. I don’t write my feedback for someone who can’t change anything or improve anything.

P20, Marginally concerned

If I share my data with the doctor, the administrator will not benefit me if they look into my data. Anyone who’s not really involved with the service. If I want to share my information in my smartphone, I want to give it to the doctor directly...It’s also the benefit of the smartphone, it can give it directly to the doctor.

P15, Marginally concerned

##### Loss of Information Agency

Participants expressed concern that collected data may be used for purposes beyond what was initially intended or disclosed, specifically that it might affect their “information agency.” This differed from their privacy concerns, where privacy risk is defined as the potential loss of personal information without the consumers’ knowledge following the use of a service or a product [23]. The loss of information agency is the loss of control over the interactions after the information has been shared by the participant. An example of loss of information agency might relate to receiving marketing emails following a service encounter, using contact information a patient provided to an endorsed third party collecting a trusted provider’s service quality survey data. In addition, 1 participant explained that the loss of agency can be apparent when seeing Web-based advertisements tailored to her daily life:

Maybe it’s stuff you don’t necessarily want a third party to know and they do know it because sometimes certain third party companies display ads based on what you’ve done if you see an ad that’s something related to you in story that you’ve done.

P21, Amateur

In general, the participants exhibited comments and knowledge consistent with their Dupree classification.

## Discussion

### Principal Findings

Mobile apps are increasingly being used to gather real-time clinical and ecological patient data and to help manage workload in the health services sectors [50-54]. Although smartphones are changing the way we deliver health care and engage patients [55], examples of their use in collecting in situ patient service experience data are scant. We explored perceptions and attitudes, which impact the adoption and use of smartphone-based apps to collect patient experience data, referred to as smart surveys. The theory of perceived risk [21,22] suggests there will be inhibitors to smart surveys adoption. Yet, although participants mentioned perceived risks normally associated with electronic commerce and other Web-based activities such as social, psychological, physical, financial, and time risks [23,56], these were considered minimal.

The study used an app that participants would have to download, retain on their phone, and manage alerts and the software app itself over time. The most commonly perceived risk was PAS, consistent with other mHealth and wearables literature [51,57,58]. Participants’ PAS risk perceptions were related to connectivity, data sharing, encryption, and storage. Consistent with Dupree et al [37], we observed that not all digital natives have the same level of technical knowledge. Our thematic analysis revealed other factors indirectly related to risk that influenced participant’s perceptions of smart surveys as a conduit for patient service experience data collection. In particular, participants trusted the data collectors and communication channel, thereby reducing perceptions of risk. Conversely, concerns over loss of information agency, evoked based on past social and personal experiences where they lost control of their data, served to amplify their PAS concerns. These themes have implications for the design and use of smart surveys apps in practice in 3 main areas. The 3 themes, with recommendations for developers are as follows:

#### Support Communication Between Providers and Recipients of Information

When individuals do trust their health care provider, the presence of trust reflects a belief that the provider has the ability and motivation to make changes that result in service improvements [59,60]. However, existing surveys often do not allow for participants to see the impact of their feedback or for providers to acknowledge its importance. Participants’ concerns revealed a need to better communicate patient experience survey goals to patients and to ensure that feedback impacts service improvements. Only half of the participants believed their feedback was important, and some participants regarded feedback as a formality rather than a tool to improve services. Similarly, we found that participants wanted health care providers who receive their comments to have the authority to implement changes. Our results point to an opportunity for smart surveys’ functionality to be expanded to include frictionless feedback loops where health care providers acknowledge the importance of participation and communicate when service feedback has been received and implemented. These communications are essential in building trust between patients and providers and are poorly supported through paper surveys.

Furthermore, our results suggest a need to better identify any complementary uses and recipients of survey data. Research ethics standards of practice require that researchers inform participants how data will be used at the beginning of a survey; however, this is not necessarily the case for private or nonprofit health service providers. Outside of personal health information, the use and management of which is often governed by legislation, consumers have very little control over what data are stored and shared for commercial use [61]. Our results suggest that participants want health care providers to affirm who and how the feedback data will be used and its sequelae.

##### Recommendation Number 1

Smart surveys functionality should foster trust between patients and providers by identifying the recipients of feedback data and communicating when it is read and what improvements to care are made as a result.

#### Provide Transparency of Motives and Options

Participants perceived a lack of confidence in the security of smartphones and that they can be perceived as a second-class computing device when compared with desktop personal computers for completing surveys. For example, participants expressed concerns about installing apps on their personal devices and uncertainty about how data may be shared between different apps. This was notable among the amateurs and marginally concerned. For individuals with more in-depth technical knowledge such as the fundamentalists, disclosure of implementation details was equally important, such as the types of permissions the app required, the type and location of servers on which the data would be stored, whether information would be encrypted, and the length of time their information would be retained. However, it should be noted that this was the exception. As with Gkioulos et al, we found that digital natives tended to ignore or were complacent about privacy policies [50].

##### Recommendation Number 2

For technical users, provide optional information about where data will be stored, for how long, and whether it will be encrypted.

##### Recommendation Number 3

Where possible, smart surveys should provide optional modalities to complete experience surveys on devices other than smartphones.

#### Controlling Access to And Sharing of Information

Although there were disagreements about the sensitivity of feedback data, participants were consistently hesitant about unauthorized use of or access to data, particularly their location (ie, GPS) [16,62]. Participants believed that location data introduced a higher level of risk and loss of agency that could lead to subsequent inconveniences (eg, telemarketing interactions) or consequences (eg, identity theft). In some cases, participants believed that loss of information agency could lead to the loss of privacy, loss of finances, or physical harm.

The perceived risk of losing agency represents a significant barrier to adoption of “advanced” smart surveys features such as geofencing (use of technology to create a virtual geographic boundary that triggers or alerts when a mobile device enters or leaves the area). For example, smart surveys can reduce recall bias by prompting patients for feedback soon after they leave a physician’s office, instead of days or months later in traditional survey methods. The majority of participants found location-based prompts too intrusive and risky and had location services disabled on their phone. This finding is consistent with prior research that demonstrates concerns for privacy are higher when the service is based on tracking the user’s location [62]. Furthermore, participants were conscious of the consequences of their location being disclosed to third parties.

##### Recommendation Number 4

Smart surveys should support alternatives to location services for prompting patients for feedback, for example, quick response codes or calendar integration.

### Limitations

The themes identified through our interviews helped to develop an understanding of barriers to smartphone-based patient experience surveys. Nevertheless, we are careful to acknowledge limitations to this study. First, the attitudes and perceptions of risk held by our participants were captured at 1 point in time, and attitudes toward adoption can change over time and as the y become more familiar with technology [63,64]. Furthermore, participants were not asked to download and use the app on their own devices. Consequently, there may have been less consideration given to risk as participants did not actually surrender any personal information. Future work will address these limitations through data triangulation and a longitudinal validation that patient behaviors reflect their reported perceptions. Second, participants were mostly younger (average age: 23.9 years) and more educated, and as a result, they are not necessarily representative of the largest patient segment using health care services [65]. Although the results of this study may not be generalizable to the wider population, a majority of adults now own cell phones, 77% of them own smartphones, and a growing percentage of adults aged 50 to 64 years and over 65 years are smartphone users (73% and 46%, respectively) [66]. These rapidly growing rates and the decreasing availability of cell phones without expanded digital capability and access to the internet suggest that adults across the life stage will soon experience similar issues as the study sample.

We used nonprobability convenience sampling and a nonsystematic recruitment process for this exploratory study; we did not anticipate our findings would be exhaustive; however, we believe that they add to the understanding of this emerging domain. On the basis of our findings, we believe that individuals with higher technical knowledge and motivation to protect their privacy were under-represented. Finally, the strength of qualitative research is its ability to describe and understand both obvious and latent phenomena contained within the “thick descriptions” provided by interview data. Although our interpretation of these exploratory data is nongeneralizable, the use of in-depth interview methodology provides researchers with an appreciation of the complexity and context of this relatively new research domain. It should also be acknowledged that with every new innovative technology, the patterns of risk and security concerns may differ from those of ostensibly similar legacy systems [24].

### Conclusions

The use of smartphone-based patient experience surveys provides new and exciting opportunities for health care providers to assess and improve the quality of health services. We conducted 24 semistructured interviews with smartphone users to explore the types of perceived risks that may exist when using smart surveys in the context-sensitive health services sector. The results demonstrate that the classical dimensions of perceived risk raised minimal concerns for the use of smartphones to collect patient service experience feedback. However, PAS risk associated with trust in the doctor-patient relationship, the reliability of the communication channel, and the risk of potential loss of agency over shared information may inhibit adoption. Conversely, altruistic motivations to contribute to health service quality for others may facilitate patients’ adoption of smart surveys. We conclude that barriers and enablers of adoption of novel technologies may change from sector to sector and should be further explored.

## Appendix

Multimedia Appendix 1 Questionnaire: participant perspectives on privacy and security.

Multimedia Appendix 2 Questionnaire: perceived risk associated with the use of smart surveys.

Multimedia Appendix 3 In-depth interview (Think Aloud) prompts.

## Abbreviations

GPS: Global Positioning System
IS: information system
mHealth: mobile health
PAS: privacy and security
