# Supplementary material for: What Is Being Used and Who Is Using It: Barriers to the Adoption of Smartphone Patient Experience Surveys
Source: JMIR Form Res. 2019 Mar 18;3(1):e9922. doi: 10.2196/formative.9922 (PMC6441859; doi:10.2196/formative.9922)
Supplement: Multimedia Appendix 1 [file formative_v3i1e9922_app1.pdf]

APPENDIX 1
Questionnaire: Participant Perspectives on Privacy and Security

Participants were asked to circle a response for each question on a 4 point Likert scale: Strongly Agree, Somewhat Agree, Somewhat Disagree, Strongly Disagree

Q1. Consumers have lost all control over how personal information is collected and used by companies.

Q2. Most businesses handle the personal information they collect about consumers in a proper and confidential way.

Q3. Existing laws and organizational practices provide a reasonable level of protection for consumer privacy today.

Q4. I have a good understanding of technical terms such as “cookies”, “encryption”, “certificate”, “Firewall” and “WPA/2”.

Q5. I am familiar with the “Terms of Use” of the online services I sign up for.

Q6. I have a good understanding of how to use the basic functions on my smartphone.
