# Supplementary material for: What Is Being Used and Who Is Using It: Barriers to the Adoption of Smartphone Patient Experience Surveys
Source: JMIR Form Res. 2019 Mar 18;3(1):e9922. doi: 10.2196/formative.9922 (PMC6441859; doi:10.2196/formative.9922)
Supplement: Multimedia Appendix 2 [file formative_v3i1e9922_app2.pdf]

APPENDIX 2

Questionnaire: Perceived Risk Associated with the Use of Smart Surveys

Following a demonstration of the MetricWire application and its features, and smart surveys, participants were asked to respond to the following questions using a 4 point Likert scale: Very Unlikely, Unlikely, Likely, Very Likely:

Q7. What are the chances that you stand to lose money if you use this smart survey (because it won't work at all, possibility of fraud or because it costs more than it should to keep it in good shape)?

Q8. What is the likelihood that there will be something wrong with smart surveys, or that it will not work properly?

Q9. What are the chances that smart surveys may not be safe; i.e. (may be or may become harmful or injurious to your health)?

Q10. What are the chances that smart survey use will not fit in well with your self-image or self-concept (i.e., the way you think about yourself)?

Q11. What are the chances that the smart survey application will affect the way others think of you?

Q12. What are the chances of possible time loss from having to set-up and learn how to use this smart survey?

Q13. On the whole, considering all sorts of factors combined, about how risky would you say it would be to sign up for and use smart surveys?
