# Supplementary material for: What Is Being Used and Who Is Using It: Barriers to the Adoption of Smartphone Patient Experience Surveys
Source: JMIR Form Res. 2019 Mar 18;3(1):e9922. doi: 10.2196/formative.9922 (PMC6441859; doi:10.2196/formative.9922)
Supplement: Multimedia Appendix 3 [file formative_v3i1e9922_app3.pdf]

APPENDIX 3

In-Depth Interview (Think Aloud) Prompts

Participants were provided with information on downloading the MetricWire application, and the potential use of the smart survey application in measuring patient experience in healthcare. Participants were asked to think about their last visit to a physician's office where they received a service, and complete a patient experience survey smart survey using the application on a researcher-provided smartphone. Interview prompts were used during and after the participants completed the experience survey and included:

Prompt: What do you think about the application so far?

Follow-Up Prompt: Are there things you like, things you dislike, things you think should be improved? Do you have any questions about the application?

Prompt: Have you ever used your smartphone to complete a survey before? (either SMS, voice-based, app, browser)

Follow-Up Prompt: What kind of survey was it? What were some reasons that you decided to/not to use your smartphone? Is providing feedback to service providers something that is important to you? Why or why not? Are there certain surveys that you prefer to use paper for?

Prompt: What are some examples of things that you wouldn't want to share using your smartphone?

Follow-Up Prompt: Are you willing to share your location through your smartphone if it is for the purpose of service quality improvement? What about other scenarios: health promotion, entering a draw, participate in research study? (benefits)

Prompt: Have you ever been concerned about who might be receiving your patient feedback information?

Follow-Up Prompt: What types of security measures should be put in place either by yourself or service providers?

Prompt: "There are no bad outcomes when it comes to sharing patient feedback using my smartphone" – do you agree or disagree with this statement?

Follow-Up Prompt: What is your definition of "bad outcomes" when it comes to providing patient feedback using your smartphone?

Prompt: What is the bigger risk concern for you when completing this smart survey?
